# Pilot Study on the Efficacy of a Novel Questionnaire for Assessing Psychological Health in Patients with Chronic Rhinosinusitis with Nasal Polyps Treated with Biologics

**DOI:** 10.3390/healthcare13040433

**Published:** 2025-02-18

**Authors:** Simonetta Masieri, Carlo Cavaliere, Antonella Loperfido, Elona Begvarfaj, Andrea Ciofalo, Francesco Maria Primerano, Gianluca Velletrani, Marcella Bugani, Pamela Cirilli, Francesco Maria Passali, Stefano Millarelli, Gianluca Bellocchi, Stefano Di Girolamo

**Affiliations:** 1 Department of Oral and Maxillofacial Sciences, Sapienza University, Piazzale Aldo Moro 5, 00185 Rome, Italy; 2 Department of Sense Organs, Sapienza University, Piazzale Aldo Moro 5, 00185 Rome, Italy; 3 Otolaryngology Unit, San Camillo Forlanini Hospital, Circonvallazione Gianicolense 87, 00152 Rome, Italy; aloperfido@scamilloforlanini.rm.it (A.L.);; 4 Department of Clinical Sciences and Translational Medicine, Otorhinolaryngology Unit, Tor Vergata University of Rome, Via Cracovia 50, 00133 Rome, Italy; 5 Eating Disorders Unit, Asl Roma 2, CTO Hospital, Via Monza 2, 00182 Rome, Italy

**Keywords:** patient centered, patient-reported outcome measures, PROMs, quality of life, survey, mental health, chronic rhinosinusitis with nasal polyps, CRSwNP, biologics, dupilumab

## Abstract

**Background/Objectives**: Chronic rhinosinusitis with nasal polyps (CRSwNP) represents a debilitating disease with significant morbidity and decreased quality of life (QoL). The introduction of biologics in its management has allowed new therapeutic options, and Dupilumab represents the first approved biologic. This study aims to evaluate a possible relationship between the clinical response to biological therapy and mental health in patients with severe CRSwNP. **Methods**: This is a multicenter study conducted at the Otolaryngology departments of three major Italian health institutions. Participants were patients with CRSwNP treated with Dupilumab. Patients were assessed at baseline and during treatment by submitting them to a survey consisting of a dedicated questionnaire focused on psychological health and two patient-reported outcome measures (PROMs): the 22-item Sino-Nasal Outcome Test (SNOT-22) and a Visual Analogue Scale (VAS) for nasal symptoms. **Results**: 86 patients were included in the study (58 males and 28 females; mean age: 58.2 years). There was a significant improvement in both symptoms and QoL, with an enhanced psychological state observed in patients after the first administration and within the first months of therapy. **Conclusions**: This study evaluated the possible correlations between Dupilumab treatment and improvements in mental health in patients with CRSwNP, as assessed through a survey, and clinical conditions, assessed through SNOT-22 and VAS. Our findings showed that Dupilumab not only improved clinical symptoms but also had a positive impact on patients’ mental health, with benefits observed already after the first administration and the first months of therapy. This survey highlights the relevance of psychological well-being and its implications for patients with chronic diseases such as CRSwNP.

## 1. Introduction

Chronic rhinosinusitis with nasal polyps (CRSwNP) is a widespread medical condition characterized by persistent inflammation of the nasal mucosa and paranasal sinuses lasting more than 12 weeks, along with the presence of nasal polyps [1]. It affects approximately 4% of the global population, with a 3% prevalence reported in Europe [2]. CRSwNP is a debilitating disorder associated with significant morbidity, a marked reduction in quality of life (QoL), and substantial healthcare and productivity costs [3].

QoL represents a general term that integrates different dimensions of life, such as physical, emotional, cognitive, psychological, economic, and social functions. A disturbance in one of these aspects, in turn, affects the other domains and influences the overall QoL [4]. Measures of QoL have evolved as the focus on medical care has shifted from objective test results and symptom scores to an analysis of the patient-centered effect of disease and response to treatments [5]. In particular, health-related quality of life (HRQoL) is defined in the literature as an evaluation domain of QoL that depends on the individual’s perception of their own health status [6]. Several authors state that the HRQoL assessment helps define the effects of chronic disease on the individual and how it interferes with his daily life. Moreover, assessing HRQoL has been shown to improve patient-physician communication and clinical outcomes [7].

HRQoL is self-determined by the individual and includes several dimensions of life that health status can directly influence. The dimensions assessed consist of physical status, social status as interaction with others, functional status as the ability to clean house or prepare food, psychological and emotional status such as depression and ability to concentrate, and finally, overall QoL [8]. Interestingly, several authors found an association between CRS and an increased incidence of anxiety and depression [9,10,11,12].

Patients with CRSwNP, due to this inflammatory state, typically suffer from persistent symptoms such as nasal obstruction, nasal discharge, facial pressure, and hypo/anosmia that significantly impact QoL. In addition, these patients may be affected by sleep disorders, depression, anxiety, and social dysfunction, which could further contribute to aggravating both their physical and mental state. Patients with CRSwNP often experience frustration due to chronic symptoms, particularly the loss of smell. The condition significantly affects various aspects of QoL, including physical health, mental well-being, work capacity, and social and emotional functioning. The impact is further exacerbated by a significant clinical burden. This burden includes increased physician visits, comorbidities such as asthma, bronchiectasis, and nonsteroidal anti-inflammatory drug exacerbated respiratory disease (NSAID-ERD), and higher healthcare costs compared to the general population. Moreover, approximately one-third of CRSwNP patients fail to achieve adequate control of their condition with standard-of-care (SoC) treatments. In particular, the SoC therapeutic strategies for CRSwNP include saline rinses, long-term intranasal corticosteroids (INCS), short courses of systemic steroids, and sinonasal surgery, such as functional endoscopic sinus surgery (FESS) and endoscopic sinus surgery (ESS), in cases of medical treatment failure [13,14]. While some patients experience improvements in both symptoms and QoL, others, particularly those with more severe manifestations, derive only short-term benefits from SoC, with nasal polyps frequently recurring after surgery. The recurrence of nasal polyps often requires additional multiple surgical procedures, with an increasingly high risk of peri-operative complications for these patients. Specifically, patients with both CRSwNP and asthma may present with more severe symptoms of either or both conditions and exhibit higher rates of nasal polyps’ recurrence, revision surgeries, and greater reliance on corticosteroids [15].

In most patients, CRSwNP is related to type 2 airway inflammation, an inflammatory pattern characterized by a peculiar subset of CD4+ T cells known as Th2 cells that secrete type 2 cytokines such as interleukin-4 (IL-4), interleukin-5 (IL-5), and interleukin-13 (IL-13), by high levels of IgE and increased eosinophils [16]. Many inflammatory diseases are associated with high levels of eosinophils and IgE [17]. Specifically, the type 2 inflammatory pathway is associated with inflammatory diseases that include not only CRSwNP but also asthma, atopic dermatitis, eosinophilic esophagitis, and NSAID-ERD [18]. As a result of this, patients with CRSwNP often concomitantly suffer from one of these comorbid conditions, particularly asthma and/or NSAID-ERD, which determines a greater disease burden [19].

As previously discussed, CRSwNP significantly impacts QoL, particularly in those patients with comorbid asthma or who require multiple steroid treatments and/or repeated surgical procedures to relieve uncontrolled symptoms [20,21,22,23]. Consequently, several authors have highlighted that CRSwNP negatively impacts the QoL in a manner that could be assumed comparable with other chronic debilitating diseases such as diabetes, chronic obstructive pulmonary disease, and congestive heart failure [24].

Monoclonal antibodies (mAbs) like Omalizumab (anti-IgE), Mepolizumab (anti-IL5), and Dupilumab (anti-IL4 receptor alpha), which target type 2 immune mediators, offer new therapeutic options in managing patients with this challenging disease [25,26,27].

Among these biologics, Dupilumab is the first approved monoclonal antibody (mAb) for the treatment of adult patients with severe CRSwNP, in addition to topical treatment with INCS, for cases that remain uncontrolled despite oral steroids and/or surgery. Dupilumab is a fully human mAb that inhibits both IL-4 and IL-13 cytokines by specifically binding to the alpha subunit of the IL-4 receptor, which is shared by both the IL-4 and IL-13 receptor complexes. Besides CRSwNP, it is used in the treatment of several type 2 inflammation-related diseases, including atopic dermatitis, asthma, eosinophilic esophagitis, and prurigo nodularis [28].

The primary endpoint of the study was to evaluate the efficacy of Dupilumab in treating severe, uncontrolled CRSwNP, focusing on symptom improvement and QoL, as assessed through the SNOT-22 and VAS for nasal symptoms. Therefore, we investigated the potential relationship between the response to Dupilumab therapy and mental health in patients with severe CRSwNP, based on the results of a newly developed, disease-specific survey focused on mental health in these patients.

## 2. Materials and Methods

### 2.1. Research Design

This is a multicenter retrospective real-life observational study jointly conducted at the Otolaryngology-Head and Neck Surgery departments of three major health institutions in Rome: Sapienza University of Rome, San Camillo Forlanini Hospital, and Tor Vergata University of Rome. Study participants were patients with severe uncontrolled CRSwNP treated with Dupilumab, a monoclonal antibody that acts against type 2 inflammatory patterns by inhibiting IL-4 and IL-13 [29]. In accordance with AIFA (Italian drug agency) guidelines, Dupilumab was administered by subcutaneous injection of 300 mg every two weeks as adjunctive therapy to INCS in adult patients with severe CRSwNP assessed by NPS ≥ 5 or SNOT-22 ≥ 50 and unresponsive to corticosteroids and/or surgery [30]. Exclusion criteria for starting treatment were radiochemotherapy for cancer in the last 12 months, pregnancy, patients suffering from hyper-eosinophilic syndromes, and patients who refused to start the biological treatment.

### 2.2. Study Variables

Patients were assessed at baseline, before starting Dupilumab, and during treatment. Clinical outcomes were measured using SNOT-22 and VAS, while mental health was assessed through a detailed survey consisting of a dedicated questionnaire focused on psychological aspects.

Specifically, patients were evaluated at baseline (T0) and during treatment between the first and the third month of therapy (T1–T3), between 3 and 6 months of treatment (T3–T6), between 6 and 12 months (T6–T12), between 12 and 18 months (T12–T18), and between 18 and 24 months of treatment (T18–T24).

### 2.3. Procedures

The assessment of nasal symptoms and QoL was conducted using the SNOT-22 and the Visual Analogue Scale (VAS) for nasal symptoms [31,32].

SNOT-22 is constituted by 22 individual custom-designed questions regarding symptom severity and HRQoL in patients affected by CRS. The outcomes measured are divided into two categories: the first category includes physical symptoms (items 1–12) comprising nasal symptoms (items 1–8) and auricular and facial symptoms (items 9–12); the second category includes health and QoL (items 13–22), which cover sleep function (items 13–16) and psychological problems (items 17–22). The total score can range from 0 to 110. A score greater than 50 is suggestive of severe nasal inflammatory disease [33].

VAS evaluates the intensity of specific symptoms, measured with a scale of values ranging from 0 to 10. Evaluated symptoms included loss of smell, nasal obstruction, headache, post-nasal drip, and nasal secretion [34].

**Figure 1:**
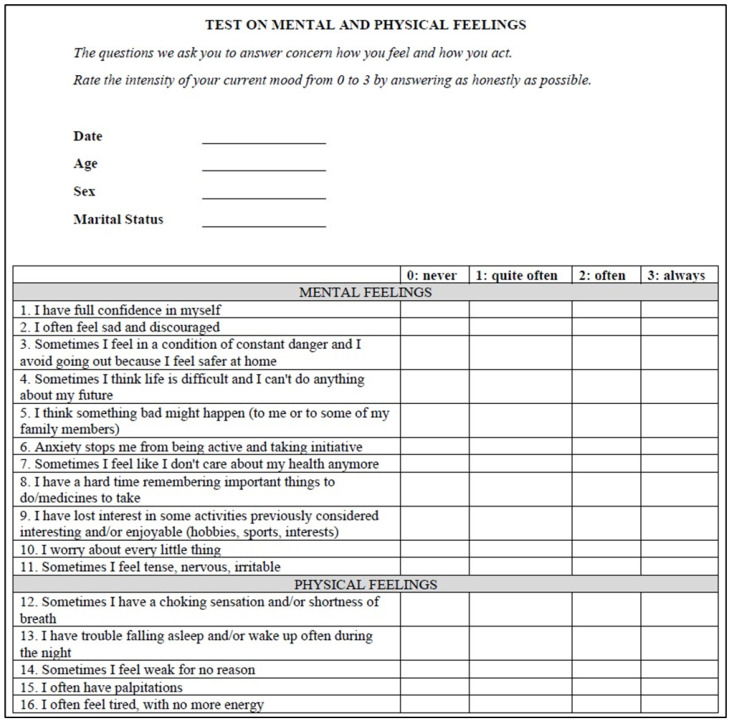
Questionnaire on mental and physical feelings in patients with CRSwNP.

In addition to evaluating clinical outcomes, this pilot study aims to determine the efficacy of a newly developed questionnaire designed to assess the psychological health of patients with CRSwNP undergoing treatment with Dupilumab. In this regard, the questionnaire that constitutes the psychological survey was created by a clinical psychologist and proposed to patients by the authors of the present paper in Italian. The concept of the survey was started in February 2024. The final survey design was completed in May 2024 after being reviewed by all authors. Medical students carried out the survey via e-mail and direct phone calls to patients undergoing treatment, and survey responses were collected up to and including October 2024. Figure 1 shows the questionnaire translated into English, comprising sixteen items subdivided into two parts: a first section (eleven questions) investigating mental sensations and a second section (five questions) assessing physical sensations. The questions cover how patients feel and act by rating the intensity of a specific sensation from 0 to 3. Except for the first question, where a high score indicates a good state of trust on the part of the patient, for all the other questions, a high score represents the maximum discomfort expressed; therefore, a score of 3 indicates severe psychological distress.

### 2.4. Data Analysis

Data from the cases included were collected and processed using the Data Analysis Tool Pak loaded in Excel (Microsoft^®^) and IBM SPSS Statistics 29.0 to calculate the statistics. A non-parametric Wilcoxon signed-rank test was used to assess the mean difference pre-post therapy among the survey, SNOT-22, and VAS. Statistical significance was assumed for *p*-values < 0.05. Spearman’s rho correlation was performed to assess the association between SNOT-22 and VAS before and during treatment.

### 2.5. Ethical Considerations

The study was conducted in accordance with the 1996 Helsinki Declaration; the Ethics committee approval was achieved (Prot. N 411/CE Lazio1 19 April 2022), and informed consent on privacy and utilization of clinical data was obtained from patients at the time of their collection.

## 3. Results

### 3.1. Sample Characteristics

A total of 86 patients were included in the study. Fifty-eight were males, and 28 were females, showing a male prevalence with an F:M ratio of 1:2. The mean age was 58.2 years (ranging from 19.8 years to 88.6 years).

A total of 76% of patients had concomitant asthma, and in 68% of cases, there were concomitant allergies, in particular for *Parietaria judaica* and dust mites. In addition, 18% of patients suffered from NSAID intolerance, and 12% reported atopic dermatitis as a comorbidity. Finally, 85% of patients underwent at least one surgery before starting Dupilumab.

During follow-up, we found a significant improvement in nasal symptoms, QoL, and mental health as assessed by SNOT-22, VAS, and the survey.

### 3.2. SNOT-22 Results Regarding the Efficacy of Dupilumab on Nasal Symptoms and QoL

The significant improvement in nasal symptoms and QoL during treatment was demonstrated by the SNOT-22, as indicated by the trends observed in this questionnaire, which is commonly used in clinical practice.

**Figure 2:**
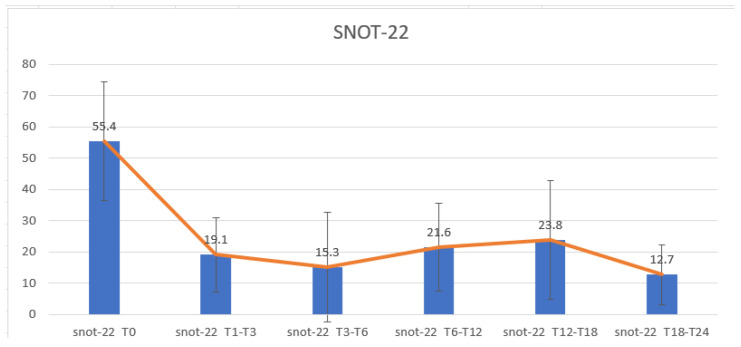
SNOT-22 trend over time. T0: Baseline; T1–T3: between 1 and 3 months of treatment; T3–T6: between 3 and 6 months of treatment; T6–T12: between 6 and 12 months of treatment; T12–T18: between 12 and 18 months of treatment; T18–T24: between 18 and 24 months of treatment.

Specifically, the SNOT-22 mean value before starting Dupilumab was 55.4 (14–109). At T1–T3, it reached the value of 19.1 (2–37). At T3–T6, the value decreased to 15.3 (1–51). After six months (T6–T12), the mean value was 21.6 (0–47); at T12–T18, the value was 23.8 (2–73); after one year and a half of Dupilumab (T18–T24), the recorded value was 12.7 (1–37). The most evident improvements occurred during the first six months of therapy at the statistical analysis. Data confirmed a significant reduction in SNOT-22 scores (*p* < 0.01). Figure 2 reports the SNOT-22 mean value trend at the different therapy periods.

### 3.3. VAS Score Results Regarding the Efficacy of Dupilumab on Nasal Symptoms and QoL

**Figure 3:**
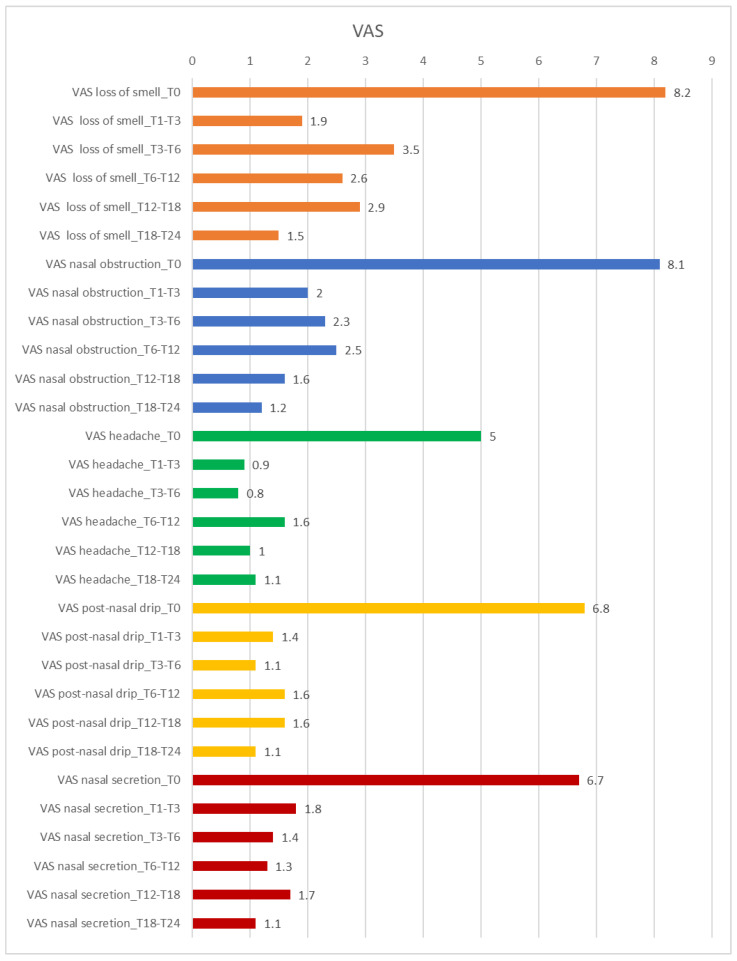
VAS trend for individual symptoms over time. T0: Baseline; T1–T3: between 1 and 3 months of treatment; T3–T6: between 3 and 6 months of treatment; T6–T12: between 6 and 12 months of treatment; T12–T18: between 12 and 18 months of treatment; T18–T24: between 18 and 24 months of treatment.

Concerning the investigated symptoms through VAS, the values for loss of smell improved from 8.2 before starting the biologic to 1.5 at T18–T24; for nasal obstruction, the main value at baseline was 8.1 and improved to 1.2 at T18–T24; for headache, the improvement went from 5 at T0 to 1.1 at T18–T24; concerning post-nasal drip, the main value at T0 was 6.8 and improved to 1.1 at T18–T24; finally, for nasal secretion, the improvement went from 6.7 to 1.1 at T18–T24. Furthermore, for all symptoms assessed at T18–T24, the *p*-value is < 0.001, an expression of high significance. Figure 3 represents the trend of each symptom analyzed on the VAS.

### 3.4. Correlation Analysis Between Parameters

**Figure 4:**
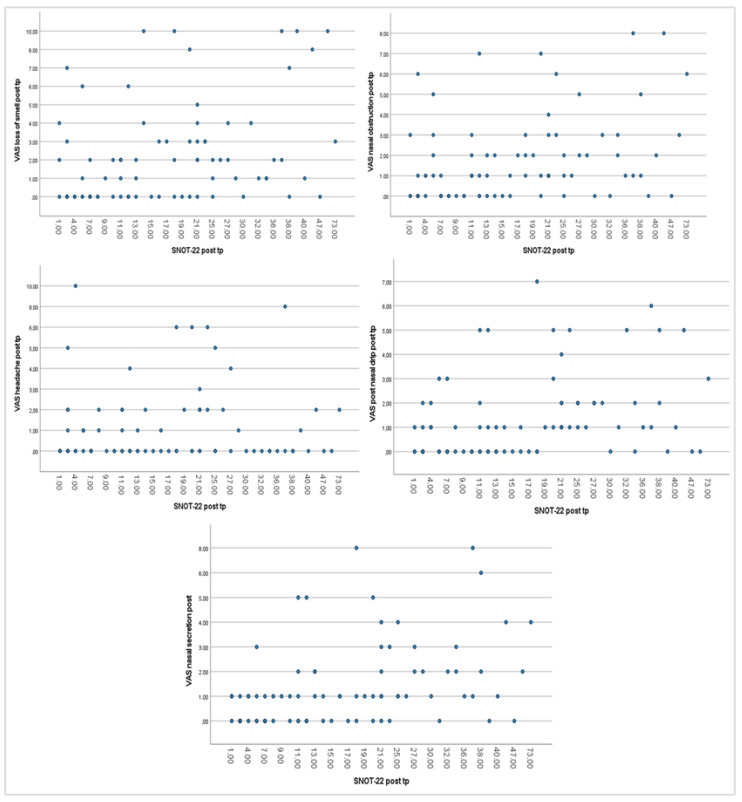
Correlations between PROMs. VAS represents the ordinate variable; the SNOT-22 represents the abscissa variable.

Figure 4 shows the correlation analysis between VAS and SNOT-22 over time from T0 to T24. The graph shows that the improvement in the VAS symptoms corresponds to an improvement in the SNOT-22.

### 3.5. Survey Results Regarding the Efficacy of Dupilumab on Psychological State

The survey showed significant differences in comparing the psychological state of patients before and during treatment. As shown in Table 1, there was an overall improvement in patients’ mental and physical feelings. Regardless of the duration of treatment, an improvement in the psychological state of the patients was observed already after the first administration and the first months of therapy.

In fact, the best results demonstrating the effectiveness of the treatment are not appreciated exclusively in the T18–T24 period. Still, they are equally distributed between the different treatment periods, demonstrating the rapid response to therapy.

## 4. Discussion

The poor QoL associated with CRS is becoming of increasing concern. In particular, from the patient’s perspective, how CRS affects daily life is much more important than the results of medical investigations such as CT scans [35]. QoL has been widely used as an essential variable to measure symptom severity and evaluate the effectiveness of therapies for CRS [36]. In particular, CRSwNP was found to have a more severe nasal symptom profile in terms of nasal discharge, nasal obstruction, and hypo/anosmia than chronic rhinosinusitis without nasal polyps (CRSsNP) [37,38]. The greatest impact on HRQoL is typical of patients with CRSwNP associated with comorbidities, including asthma, chronic obstructive pulmonary disease, NSAID-ERD, bronchiectasis, and obstructive sleep apnea, or those with rare subtypes of CRS, such as allergic fungal rhinitis [39,40,41,42]. Both symptoms and secondary consequences of CRSwNP can significantly influence HRQoL [43].

There is a growing interest among clinicians and researchers in the impact of CRSwNP and its therapeutic management on patients’ QoL. In particular, the most up-to-date international literature on the use of new biologic drugs in the therapeutic management of CRSwNP considers the improvement of the patient’s QoL as one of the parameters for evaluating treatment efficacy [44].

Specifically, to assess the response to the biologic, the authors of the most recent guidelines also consider the improvement in the patient’s QoL based on the SNOT-22 questionnaire. Among the patient-reported outcomes questionnaires (PROMs) for CRSwNP, the SNOT-22 is considered one of the most accepted and widely used questionnaires in clinical practice [45]. It represents a value that may direct clinicians to treat a patient with severe uncontrolled CRSwNP with biological therapy [33].

In addition to SNOT-22, another PROM used for CRSwNP is VAS, which measures the extent of disease-specific symptoms and, consequently, their impact on patients’ QoL. This scale examines typical symptoms of CRS, including nasal congestion, loss of smell, post-nasal drip, nasal discharge, and headache, giving greater weight to the nasal domain than SNOT-22. Specifically, VAS evaluates the intensity of these nasal symptoms, measured on a 10 cm horizontal line. A mean score for each symptom evaluated is obtained using the average value of the scores. This scale is widespread in clinical practice since it is quick and easy for the patient [46].

In our real-world experience, Dupilumab has shown to be effective in severe uncontrolled CRSwNP management, demonstrating efficacy in improving nasal symptoms and their impact on QoL, as reported at SNOT-22 and VAS. These results confirm the efficacy and safety of this biological treatment for severe CRSwNP management, which is in line with current scientific evidence [47].

Moreover, in the recent literature, there has been a growing interest in exploring patients’ experiences living with CRSwNP, and surveys have also been used as an evaluation method [48,49].

Surveys are essential tools in clinical research, particularly for assessing the QoL in patients. They provide structured methods for collecting subjective data on individuals’ mental and physical sensations, which are critical indicators of overall well-being. The implementation of surveys enables researchers to systematically evaluate various dimensions of health, including psychological, physical, and social domains. This comprehensive approach is crucial for understanding the multifaceted impacts of diseases and treatments on patients’ daily lives. The use of surveys in evaluating QoL and mental and physical sensations offers valuable insights that are integral to patient-centered care and the development of targeted interventions [50].

In this study, we aimed to assess the impact of CRSwNP on patients’ QoL using standard instruments such as PROMs and a dedicated survey. This survey consisted of a questionnaire on mental and physical sensations in patients with CRSwNP on biological therapy and allowed us to see the significant impact on these areas, often overlooked in clinical practice [51].

In this regard, patients suffering from chronic conditions such as CRSwNP may experience a wide range of emotional and psychological sensations that significantly affect their overall well-being. These emotions, which may include feelings of sadness, discouragement, constant anxiety, and a sense of impending danger, can contribute to a deterioration in their QoL. Additionally, patients may experience emotional detachment from their health, exhibit signs of depression, and suffer from fatigue and anxiety, all of which can lead to a profound sense of helplessness and exhaustion. Furthermore, it was reported that patients with CRSwNP and anosmia experienced decreased QoL and increased depression and anxiety [15]. To better understand the psychological impact of CRSwNP on our patients, we developed a dedicated survey designed to assess these emotional states. The survey focused on key aspects such as loss of self-confidence, anxiety, fatigue, irritability, phobia, and depressive symptoms. Furthermore, it aimed to evaluate whether biological therapy, specifically Dupilumab, could influence the emotional well-being of patients and contribute to improvements in their psychological state. By measuring these emotional responses, we sought to gain deeper insight into the psychological burden of CRSwNP and to determine if effective symptom management could lead to positive changes in the mental health of patients.

In our experience, patients reported an improvement in their psychological and physical state already after the first administration of therapy, which is in line with other studies [52,53]. It is widely recognized in the literature that evaluating the effectiveness of different treatments in CRS is essential, as it is a condition with increasing prevalence and cost that affects a significant portion of the population. For a comprehensive and thorough assessment of CRS morbidity and treatment effectiveness, it is essential to validly measure the physical, emotional, and social problems associated with this condition [54]. In our experience, this survey can be considered a good tool for this purpose.

This is a pilot study intending to determine the efficacy of a newly developed questionnaire characterizing the psychological health of patients affected by CRSwNP in therapy with Dupilumab and, as such, represents some limitations. First, the retrospective nature of the study. Second, the number of patients. Additionally, the follow-up period was limited to 24 months, which, although considerable compared to other published series, is insufficient to draw conclusive evidence. Furthermore, the data were self-reported, which may have led to bias, as patients with greater disease severity might have been more inclined to participate in the survey. Finally, patients were interviewed at different therapy timelines when the questionnaires were administered, and we chose to evaluate the effect of Dupilumab on psychological health and QoL over time.

This study examined the link between Dupilumab treatment and improvements in both mental health and clinical symptoms in patients with CRSwNP. Clinical outcomes were measured using SNOT-22 and VAS, while mental health was assessed through a detailed survey. The results showed that Dupilumab not only improved clinical symptoms but also had a positive impact on patients’ mental health, with benefits observed early in the treatment process. The detailed and complex questions in the survey provided valuable insights into the psychological challenges faced by patients with chronic conditions like CRS. These findings emphasize the importance of addressing both physical and mental health in the treatment of CRSwNP, demonstrating that effective therapy can enhance overall well-being. Further research is required to validate and standardize the questionnaire, ensuring its clinical applicability and broader use in patient assessments.

## 5. Conclusions

This study evaluated the possible correlations between Dupilumab treatment and improvements in patients with CRSwNP in both mental health, assessed through a survey, and clinical conditions, assessed through SNOT-22 and VAS. Our findings showed that Dupilumab not only improved clinical symptoms but also had a positive impact on patients’ mental health, with benefits observed already after the first administration and the first months of therapy. This survey highlights the relevance of psychological well-being and the implications for patients affected by chronic diseases such as CRS.

## Figures and Tables

**Table 1:** Survey Results. T0: Baseline; T1–T3: between 1 and 3 months of treatment; T3–T6: between 3 and 6 months of treatment; T6–T12: between 6 and 12 months of treatment; T12–T18: between 12 and 18 months of treatment; T18–T24: between 18 and 24 months of treatment. SD: standard deviation.

Question		T0	T1–T3	T3–T6	T6–T12	T12–T18	T18–T24
1. I have full confidence in myself	Mean	1.91	1.9	1.5	2.36	2.21	2.51
	SD	1.1	1.0	1.3	1.0	1.1	0.9
2. I often feel sad and discouraged	Mean	1.01	0.7	1.13	0.27	0.29	0.23
	SD	1.1	0.7	1.4	0.5	0.6	0.6
3. Sometimes I feel in a condition of constant danger and I avoid going out because I feel safer at home	Mean	0.41	0.1	0.63	0	0.14	0.05
	SD	0.8	0.3	1.1	0.0	0.5	0.2
4. Sometimes I think life is difficult and I can’t do anything about my future	Mean	0.64	0.7	0.75	0	0.36	0.05
	SD	1.1	1.3	1.4	0.0	0.6	0.2
5. I think something bad might happen (to me or to some of my family members)	Mean	0.56	1	0.5	0.09	0.29	0.23
	SD	0.9	0.7	0.5	0.3	0.8	0.6
6. Anxiety stops me from being active and taking initiative	Mean	0.62	0.1	0.38	0.09	0.64	0.16
	SD	0.9	0.3	0.7	0.3	0.8	0.4
7. Sometimes I feel like I don’t care about my health anymore	Mean	0.24	0	0.13	0	0	0.05
	SD	0.6	0.0	0.4	0.0	0.0	0.2
8. I have a hard time remembering important things to do/medicines to take	Mean	0.34	0.3	0.5	0.27	0.21	0.16
	SD	0.7	0.5	1.1	0.6	0.6	0.5
9. I have lost interest in some activities previously considered interesting and/or enjoyable (hobbies, sports, interests)	Mean	0.84	0.5	0.25	0.27	0.29	0.33
	SD	1.0	0.7	0.5	0.6	0.5	0.6
10. I worry about every little thing	Mean	0.77	0.91	0.71	0.36	0.29	0.4
	SD	1.0	0.9	1.1	0.5	0.5	0.8
11. Sometimes I feel tense, nervous, irritable	Mean	1.24	1.1	0.75	0.45	0.64	0.44
	SD	1.0	1.1	0.9	0.7	0.7	0.7
12. Sometimes I have a choking sensation and/or shortness of breath	Mean	1.31	0.5	0.63	0.45	0.57	0.14
	SD	1.1	0.9	0.7	0.7	0.6	0.4
13. I have trouble falling asleep and/or wake up often during the night	Mean	1.53	1.3	1	0.82	0.64	0.44
	SD	1.1	1.3	1.3	1.0	0.9	0.7
14. Sometimes I feel weak for no reason	Mean	1.08	0.5	0.5	0.27	0.93	0.26
	SD	1.0	0.7	0.8	0.6	1.1	0.5
15. I often have palpitations	Mean	0.67	0.9	0.13	0.18	0.57	0.23
	SD	0.9	0.9	0.4	0.4	0.8	0.6
16. I often feel tired, with no more energy	Mean	1.22	1.2	0.63	0.55	0.71	0.3
	SD	0.975	0.632	0.916	0.688	1.069	0.558

## Data Availability

The original contributions presented in this study are included in the article. Further inquiries can be directed to the corresponding author(s).

## Abbreviations

The following abbreviations are used in this manuscript:

CRSwNP	Chronic Rhinosinusitis with Nasal Polyps
QoL	Quality of Life
IL	Interleukin
IgE	Immunoglobulin E
NSAID-ERD	Nonsteroidal Anti-Inflammatory Drug-Exacerbated Respiratory Disease
INCS	Intranasal Corticosteroids
Mabs	Monoclonal antibodies
CRS	Chronic Rhinosinusitis
AIFA	Italian Agency of Drugs
NPS	Nasal Polyp Score
SNOT-22	22-item Sino-Nasal Outcome Test
VAS	Visual Analogue Scale
T	Time
M	Male
F	Female
HRQoL	Health-Related Quality of Life
CT	Computed Tomography
CRSsNP	Chronic Rhinosinusitis without Nasal Polyps
PROMs	Patient-Reported Outcome Measures
